# Structure, functions, performance and gaps of event-based surveillance (EBS) in Sudan, 2021: a cross-sectional review

**DOI:** 10.1186/s12992-022-00886-6

**Published:** 2022-12-01

**Authors:** Elfatih Mohamed Malik, Ahmad Izzoddeen Abdullah, Sabir Ali Mohammed, Abdelgadir Ali Bashir, Rayyan Ibrahim, Abdalla Mohammed Abdalla, Muntasir Mohamed Osman, Tahani Amin Mahmoud, Mohamed Abdalhafiz Alkhidir, Suleiman Gamal Elgorashi, Mazza Abasher Alzain, Omer Elbadri Mohamed, Ismaiel Mohamed Ismaiel, Hatim Fadelalsyeed Fadelmula, Babiker Ahmed Ali Magboul, Muzhgan Habibi, Mahmoud Sadek, Ahmed Aboushady, Christopher Lane

**Affiliations:** 1 GHD|EMPHNET, Khartoum, Sudan; 2 Faculty of Medicine, University of Khartoum, Khartoum, Sudan; 3 Sudan FETP, Federal Ministry of Health, Khartoum, Sudan; 4 Khartoum, Sudan; 5 FMOH, HEEC, Khartoum, Sudan; 6 Surveillance Officer, WHO Office, Khartoum, Sudan; 7 WHO Regional Office for the Eastern Mediterranean, Cairo, Egypt; 8 WHO Country Office, Khartoum, Sudan

**Keywords:** Surveillance, Event-based surveillance, Community-based surveillance, Health signals, Case detection, Epidemic response, Epidemic control

## Abstract

**Background:**

Event-based surveillance (EBS) is an essential component of Early Warning Alert and Response (EWAR) as per the International Health Regulations (IHR), 2005. EBS was established in Sudan in 2016 as a complementary system for Indicator-based surveillance (IBS). This review will provide an overview of the current EBS structure, functions and performance in Sudan and identify the gaps and ways forward.

**Methods:**

The review followed the WHO/EMRO guidelines and tools. Structured discussions, observation and review of records and guidelines were done at national and state levels. Community volunteers were interviewed through phone calls. Directors of Health Emergency and Epidemic Control, surveillance officers and focal persons for EBS at the state level were also interviewed. SPSS software was used to perform descriptive statistical analysis for quantitative data, while qualitative data was analysed manually using thematic analysis, paying particular attention to the health system level allowing for an exploration of how and why experiences differ across levels. Written and verbal consents were obtained from all participants as appropriate.

**Results:**

Sudan has a functioning EBS; however, there is an underestimation of its contribution and importance at the national and states levels. The link between the national level and states is ad hoc or is driven by the need for reports. While community event-based surveillance (CEBS) is functioning, EBS from health facilities and from non-health sectors is not currently active. The integration of EBS into overall surveillance was not addressed, and the pathway from detection to action is not clear. The use of electronic databases and platforms is generally limited. Factors that would improve performance include training, presence of a trained focal person at state level, and regular follow-up from the national level. Factors such as staff turnover, income in relation to expenses and not having a high academic qualification (Diploma or MSc) were noticed as inhibiting factors.

**Conclusion:**

The review recommended revisiting the surveillance structure at national and state levels to put EBS as an essential component and to update guidelines and standard operation procedures SOPs to foster the integration between EBS components and the overall surveillance system. The need for strengthening the link with states, capacity building and re-addressing the training modalities was highlighted.

## Background

Surveillance is the process of a continuous and systematic collection, analysis, interpretation and dissemination and the use of this data for action [1]. Surveillance systems vary in tools, scope, goals, and characteristics, and what is considered important in one country could be less important in another [2]. Therefore, when structuring a system, careful balancing should be done to ensure system flexibility. The aim as stated by Shahab, must be ‘‘adapt not adopt’’ [3]. With emerging and re-emergent infectious diseases and the adoption of international health regulations, communicable disease surveillance has become widely adopted in developing countries [4, 5] and is becoming a cornerstone in control of diseases and events. Communicable disease surveillance consists of core and supportive activities. The core activities include case detection, registration, laboratory confirmation, data reporting, data analysis, feedback, and epidemic preparedness and response. The supportive activities include coordination, supervision, training, and mobilization of resources [6].

The communicable disease surveillance (indicator-based surveillance- IBS) in Sudan is sentinel-based and it can be classified as passive surveillance that shifts into partially active during epidemics or outbreaks [7]. For the passive surveillance, 30% (1918/ 6393) of public health facilities report on a weekly basis and measures to include more health facilities were recently adopted by the Ministry of Health and its partners. During epidemics (e.g., COVID-19) or when the risk of occurrence is high, the Surveillance and Information Department (SID) under the Health Emergency and Epidemic Control (HEEC) General Directorate at the national level expands the surveillance system to cover all the health facilities and requires daily reporting even if there are no cases (zero reporting). The Federal Ministry of Health (FMOH) applied this daily reporting system during COVID-19; but as there is no system to track facilities that report zero cases, its usefulness was uncertain. A study assessing surveillance activities and functions conducted in Khartoum state in 2010 concluded that, despite good knowledge and data reporting, there was poor data analysis, preparedness, feedback to reporting facilities, documentation, and system updates [8]. The national surveillance system was also found to be not representative, as it did not include the private, military, and teaching hospitals and facilities.

Event-base surveillance (EBS), defined as the “*Organized collection, monitoring, assessment and interpretation of mainly unstructured *ad hoc* information regarding health events or risks, which may represent an acute risk to human health,*” gained attention following the revision of the International Health Regulations (IHR) (2005) which expanded usual infectious disease notification to include surveillance of public health events of various origins. Furthermore, the IHR urges countries “…*to strengthen and develop both routine, indicator-based, surveillance and event-based surveillance*” [9, 10]. EBS is especially needed where coverage with indicator-based surveillance is limited, and lessons learned from the Ebola outbreak in West Africa and its associated challenges highlight this as an urgent issue [11]. In 2014, the World Health Organization (WHO) developed a guide for the implementation of early warning system with focus on EBS [12].

In Sudan, EBS was established in 2016 with guidelines and Standard Operational Procedures (SOPs) for implementation. This was followed by training of EBS focal persons at state level with the training in some states extending to the community level as part of community event-based surveillance (CEBS). EBS is currently considered an important supportive activity for SID, and it also provides support to the Emergency Operation Centre (EOC) for decision making. The need for an assessment of core activities (detection, registration reporting and analysis) and supportive functions (coordination, supervision, training and resources mobilization) [11] of EBS in Sudan was raised by the HEEC Director in order to learn from current practice and for further development of the EBS system. This review provides an overview of the current EBS structure, functions and performance in Sudan and identifies the gaps and ways forward.

## Methodology

### Study design and area

**Fig. 1:**
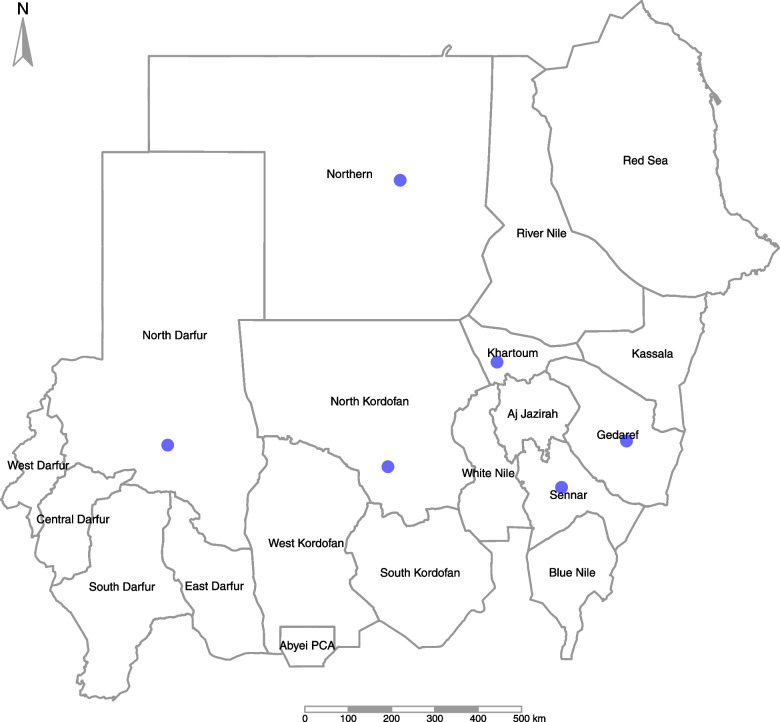
Sudan map showing the capital of the six visited states (green circle indicates the state capital)

A cross-sectional study using mixed quantitative and qualitative methods was conducted during the period from March—April, 2021. The study reviews the situation at the national level and at 6 states (out of the 18 states in Sudan) selected based on their vulnerability to epidemics considering the socioeconomic, demographic, housing and hygiene, epidemiological, and health system status. Each state represents a region of Sudan (Fig. 1).

### Data collection and sampling

The study followed WHO/EMRO guidelines and tools to assess the establishment and implementation of EBS in Sudan (World Health Organization/Earstern Mediteranian Regional Office: Manual for the assessment of the public health surveillance system with a focus on Early Warning And Response (EWAR) and Event-Based-Surveillance (EBS), unpublished). HEEC directors, surveillance officers, and focal persons for EBS, CEBS, point of entry (POE), and Incidence Tracking System (ITS) at states level were interviewed using a self-administered questionnaire disseminated through email, the total number of interviews was 53. Seven focus group discussions (FGD) were arranged, one at the national level and one for each state. The participants included the focal persons of EBS, CEBS, POE and for ITS at visited states, in addition to stakeholders and other partners (e.g., animal sector, civil defence, weather meteorology and education).

Five to seven frontline volunteers were randomly selected from a list of those who were currently involved with CEBS and interviewed via phone. The selection was done through a simple random sampling from the state registry of volunteers, the total number of volunteers interviewed was 26. The EBS structure and supporting functions at national and selected states were reviewed using forms developed by WHO/EMRO (World Health Organization/Earstern Mediteranian Regional Office: Manual for the assessment of the public health surveillance system with a focus on Early Warning And Response (EWAR) and Event-Based-Surveillance (EBS), unpublished) “*EMR Country Landscape Assessment: Integrated Disease Surveillance with a Focus on Event-Based Surveillance*”. The forms were completed through group discussion and observation of annual plans, HEEC structure, and reports (weekly, monthly and event-based reports). To assess the performance of EBS, a score sheet was developed to compile findings from the interview, review of records, and observation by the assessment team members. There were 6 teams carried out the assessment, one team was sent for each state. Each assessment team was asked to score the performance of visited states in 21 items covering aspects related to detection, reporting, verification, risk assessment, perception, planning, implementation and monitoring of EBS at states level. The maximum score for each item is 10 marks.

### Data analysis

Statistical Package for Social science (SPSS version 23) was used to analyse quantitative data where descriptive statistics was performed. Percentages were used to describe the data where appropriate. Data was presented using frequency tables. Qualitative data was organized in themes and analysed manually using a thematic analysis, paying particular attention to the health system level allowing for an exploration of how and why experiences differ across levels.

## Results

### Structure of EBS

**Fig. 2:**
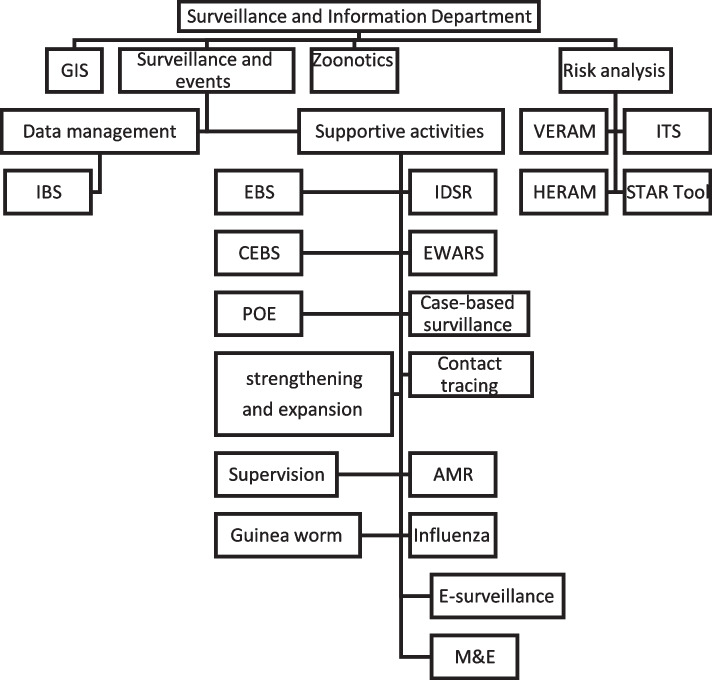
The structure of SID at national level

**Fig. 3:**
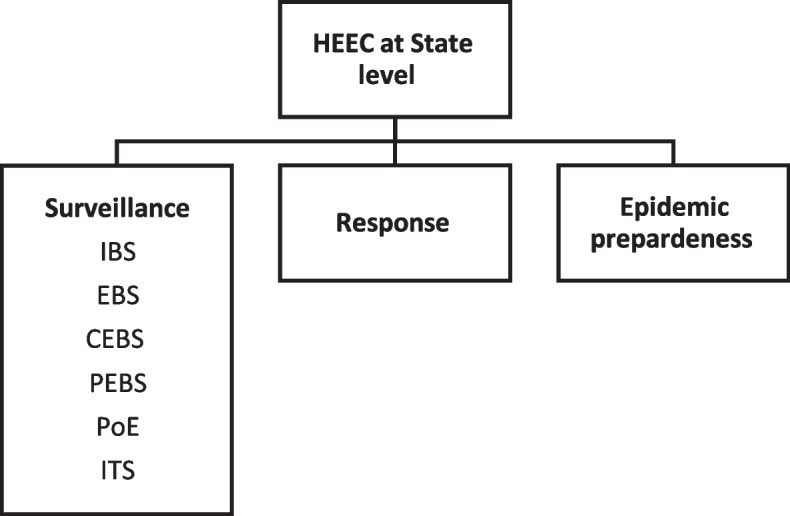
The structure of HEEC at state level

At the national level, surveillance, through the SID, is part of the HEEC General Directorate. The overall structure of the SID at national level consists of 4 sub-directorates with a unit called “Supportive Activities” where training, supervision and many other activities are listed under the umbrella of this unit (Fig. 2). The EBS components (Partner-based surveillance-PEBS, CEBS, HEBS, POE, hotline and media scanning-HMS) are under the Supportive Activities unit with a focal person for each and a coordinator for all EBS activities. There was no written job description to show the roles and responsibilities of the EBS coordinator and focal persons, but there were SOPs for each project including responsibilities at different levels. At the state level a very simple structure was adopted (Fig. 3) where IBS and EBS are under the umbrella of the Surveillance Unit with 1–3 persons responsible for the work. Overall, states give less attention to EBS as compared to IBS, and EBS at the state level is equivalent to CEBS with no attempts to implement other forms of EBS (hotline, health facility and media scanning). In fact, very few EBS trained focal persons remained at states indicating high turnover attributed to political instability and low salary.

The surveillance and epidemic control arrangements and activities are governed by the Sudan Constitution and by the National Public Health Law, 2008 in addition to IHR, 2005, and by the establishment of HEEC General Directorate at states levels (and hence surveillance and Information department) in 2014. No law, regulation or decree is designed for EBS separately, nor is there an enforcement mechanism in place to accelerate the implementation of EBS. The government contribution was limited to meet the salaries for surveillance officers and to cover the free telephone network (with paid internet) for the state and localities staff. The Ministry of Health with WHO supplied each volunteer at the community level with a simplified registries for recording signals and events and SOPs to guide the volunteers although some volunteers never received this. As well, only a small number of volunteers experienced refresher training and supportive supervision from the state EBS surveillance officers. This reflects low institutional and political commitment to the system since its establishment.

Apart from regularly scheduled coordination meetings between implementers (Federal and States MoH) and stakeholders during an epidemic or health emergencies, there was no outlined mechanism for regular coordination at national and state levels regarding EBS implementation. There was no technical working group of key implementers or broader coordination committee of partners to manage coordination at the national level. However, the Emergency Operation Centre (EOC) daily meeting (during times of public health crises) involves partners (WHO, UNICEF, Non-health sectors) and implementation bodies, and is the forum that reviews the surveillance data and response activities. No formal link between national level and states exists apart from the requirement for immediate reports for the detected signals/ events in addition to monthly reports. Ad hoc phone calls were sometimes arranged by the national level particularly when there are rumours or notification needs triage and/or verification at the state level.

The national level has developed and availed soft and printed format guidelines and SOPs for EBS (PEBS, CEBS and POE) to direct the implementation at states level. The guidelines identified the priority events and signals, defined the role and responsibilities of each level, and stated clearly the information flow. While some states currently use the SOPs and guidelines, they also reported shortages of supply of SOPs and guidelines, while other states did not know these existed.

In 2016–17, the national level trained states’ EBS focal persons together with 18–22 partners at each state. Partners at state level include governmental sectors (animal, agriculture, police, climate and meteorology, education, public mass media), civil societies, UN agencies, international NGOs, and big development schemes. These partners differed from state to state. Furthermore, all focal persons for CEBS at states level were trained in 2018 to be trainers of trainers “TOT”, disseminating more widely to community volunteers’ knowledge of the SOPs and guidelines they had received. No formal refreshment training and no regular follow-up or supportive supervision for focal persons was provided. The exact need of personnel for EBS and the target for training was not clear at both national and states levels.

EBS has its own reporting format and data flow which was partially integrated at national and states levels. At the national level, the weekly meetings foster the information sharing and coordination between the different projects as per the current structure of SID; in these meetings, both EBS and IBS reports are presented and discussed and the final decision made about the disease/ event under discussion. The EBS monthly report compiled all the signals/ events from different states is and submitted separately to the Head of the Department.

### EBS core functions

The FMOH developed signals to be detected by the EBS. The CEBS for instance is expected to detect and report unusual, unexpected signals with particular emphasis on acute respiratory symptoms, haemorrhagic fever, acute diarrhoea, jaundice, acute neurological symptoms, guinea worm, floods, draught, displacement, conflicts, and death among animals. As part of CEBS, the trained community volunteers report signals and events immediately when detected. The Ministry of Health has no unified form for reporting but the volunteer is expected to describe what is happening, where, when, who and how many affected, how many have died …etc. Each group of volunteers assigns one person to be the coordinator. When the community volunteers detected a signal, they report either to volunteer coordinator or directly to the locality level using telephone, direct contact, or through another person. The contribution of partners (including other governmental sectors like animal sector) was limited to the detection and reporting of signals to state health authorities. On some occasions, partners (e.g., animal sector) report to its relevant authority at the national level and this authority informed the national health authority. No system exists to capture rumours, official media reports about unusual or unexpected events apart from phone calls from individuals to the emergency call centres (ECC) at national and state levels (using the emergency numbers). With the exception of Gedarif state (out of 6 states visited), there were no official rumours logbooks or databases for the registration of suspected public health events from informal sources, making the follow-up of signals after detection very difficult. Efforts were ongoing to enhance follow-up based on the OSM (Online Signal Module). Some volunteers and focal persons used a notebook for registration, but it was not standardized to an official format. There are no weekly or monthly reports required from volunteers, only monthly reports from the state level). Volunteers are expected to report when there is a signal; therefore “n*o report means zero signals*!!” as stated by one surveillance officer in a state.

States report to the national level immediately when there is a signal or event. By the end of the month, states are expected to compile all reported signals and events and send to Federal MOH using a structured format which covers the what, where, when, who, and how of the signal or event. The focal points for EBS and CEBS at the national level compile reports from all states and issue their monthly reports. The contents of these reports are discussed as part of “Surveillance and Information Department” and “HEEC General Directorate” formal meetings. Few states showed a monthly EBS or CEBS reports but there are separate reports for each event. There was no attempt observed to use database for signals/ events reporting at the state level.

**Fig. 4:**
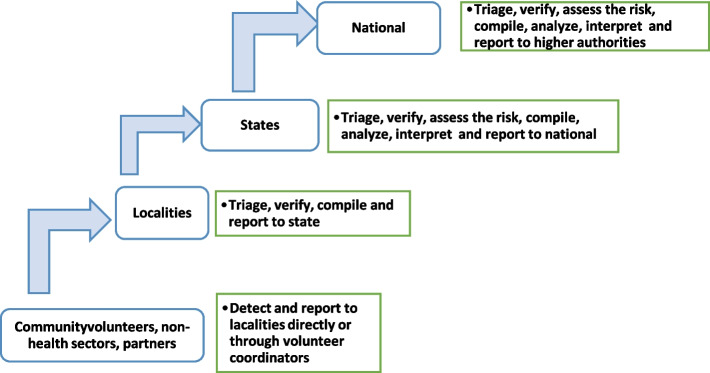
Role of each level and the information flow in EBS

Once the locality surveillance officer receives a notification from a community volunteer or another source, they inform the state and started arrangements for triage, verification, and risk assessment, if needed. This process depends on the locality resources and, in most cases, is completed jointly with the state team. The team sends a written report to the Director General of Health at the state, and if the event represents a public health event of concern, the director informs the Federal MOH. The state conducts verification, risk assessment, and response, which is carried out by trained rapid response teams (RRTs). The training process of RRTs was accelerated by the COVID-19 pandemic (Fig. 4).

### Performance of state EBS

**Fig. 5:**
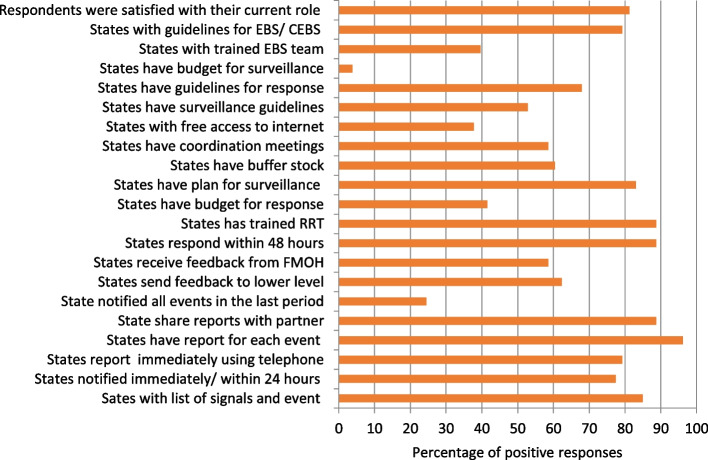
Respondents reported self-assessment related to detection, reporting, verification, risk assessment, perception, planning, satisfaction related to EBS at states level

After visiting the states and analysing study data the assessment teams gave a score for each state out of 100 expressed as a percentage. In 14 out of the 21 items, the overall score was high, more than 50, (Fig. 5). However, the assessment teams expressed concerns about the lack of a structured collaboration with partners: on most occasions there was no collaboration, or it was weak. Moreover, in 2 states there was no EBS unit or focal persons. Most states did not have a written organogram or define roles and responsibilities for EBS staff. Although the current personnel were trained in surveillance and in EBS, the trained personnel were not sure about their capability to do the assigned work. Free access to the internet was limited and supportive supervision from the state to localities and to frontline personnel was lagging (See Table 2 in the appendix).

### EBS as perceived by state surveillance officers

The above-mentioned findings were confirmed by the data obtained from 53 surveillance officers working at the state level. Around 60% of respondents reported to have a list of signals with standard definition. More than 75% of respondents stated there was a presence of a community-based system to capture an unusual, unexpected or new event. Ninety percent of the information captured was through the call centre, volunteers or health care workers (See Table 3 in the appendix).

All the 53 respondents reported detecting signals/ events in the last year (the reported information is initially a signal, but when the occurrence is verified, it is reported by them as event). Over two-thirds of the respondents stated having personal notes in which they recorded information about signals such as date, time, place, source of information, initial cause, description of the signal/ event, and number of cases/ deaths occurred as a result. Less attention was given to having a unique serial number to signal/ event (37.7%). The other important finding was that states were inadequate in the following areas: database development (54.7%), electronic system (39.6%), list of experts (47.2%) and presence of a public health laboratory (35.8%). A total of 33 (62.3%) respondents knew the recommended time for verification, but two thirds (67.9%) stated having a risk analysis and even more reported conducting risk analysis (86.8%) using Federal MOH or WHO tools. Unfortunately, only 30 out of 53 candidates were involved in the analysis of the last risk reported in their states. Generally, limited numbers of respondents (41.5%) attempted to analyse paper-based EBS and IBS data at state level as shown in Table 1.

**Table 1:** Detection, registration, verification and risk analysis of signals and events (*n* = 53)

Variables	Frequency	%
Ever detect a signal/ event at state level	45	84.9
State with a register for signals/events	38	71.7
Event has a unique serial number	20	37.7
Event notification date and time documented	40	75.5
Initial cause of the event registered	33	62.3
There is a description for the event	36	67.9
Cases and deaths reported	40	75.5
Event place stated	41	77.4
Event source of information stated	38	71.7
State has databases	29	54.7
State has e-system	21	39.6
Respondents know verification time	33	62.3
State has a list of experts	25	47.2
State has a Public Health Lab	19	35.8
State analyses data on regular basis	22	41.5
State has risk analysis team	36	67.9
State conduct risk analysis for the detected signals/ events	46	86.8
State use MOH or WHO tool for risk analysis	44	83.0
Last risk analysed	30	56.6

Respondents also reported their self-assessment related to detection, reporting, verification, risk assessment, perception, planning, implementation and monitoring of EBS at states level (Fig. 5). Many of them stated that states have a list of signals/ events for immediate notification (84.9%) and knew the time for notification (77.4%). The lower level notified to higher level (e.g., a community volunteer notifies to the locality focal person, who in turn reports to the state focal person) immediately or within 24 h using the telephone in most of the cases.. Most states reported signals and events to the national level immediately; however, limited number of states reported on weekly or monthly basis only. Telephone is the reporting tool. States have a report for each event, keep a copy of their sent reports, and share the report with the non-health sector and NGOs. Sending or receiving feedback report was not identified by reviewers as a common practice. The response to an event and to an emergency, in general, is perceived as good and findings related to supporting activities for EBS and surveillance were encouraging. More than 80% of respondents stated having plans for surveillance and response, coordination committees with partners, supported telephone calls, guidelines for CBES, and have integrated EBS into broader surveillance. Respondents expressed concerns about meeting with partners, buffer stock, and guidelines for overall surveillance for response. Few reported having free access to internet, ore awareness of HEEC being established in the state based on law or decree, or of an EBS team.

### EBS at state level as perceived by the community volunteers

Following the TOT training in 2018, states, through support from the Federal MOH and other partners, identified target areas and trained community volunteers. States keep records of the volunteers which includes telephone numbers. During the visits to five states, the assessment teams) randomly selected 5 – 7 volunteers from the list and communicated with them by phone. Surprisingly, almost all attempts succeeded. Out of 26 volunteers, 13 were female. The mean (SD) age was 37.9 (11.1) years ranging between 23 to 62 years. Fourteen have basic education and 10 have university/ above university education. Eighteen designated themselves as community volunteers and 4 as health care providers. The majority (21 respondents) were involved with CEBS for 2 years or less, and 5 of them were involved in such work for more than 5 years. Twenty-two of them were trained and knew what their role was, and almost all knew what needed to be reported. Eighteen of the respondents had reported an event before. Most of the reported events could be classified as biological (diseases) and a few were social (displacement). The events came to the attention of the volunteers during engagement in social gathering, personal contact and observation. Volunteers used phones to notify to the higher level, the locality EBS focal person. The majority of respondents at the community level identified no hindrances apart from communication network problems. Only 11 respondents reported having notification forms, and 10 had a register for events. Of those ten, only 4 registered the last event they had. One third of them received occasional feedback from the lower level. What is outstanding is that 20 were satisfied with CEBS and 22 were willing to continue. The volunteers’ suggestions to improve the project include support to phone calls, training and supplies such as notification forms and registers.

## Discussion

This mixed qualitative and quantitative cross-sectional review aimed at exploring the EBS implementation and providing insight to existing infrastructure, program gaps, rationale behind the current system, and program improvements. EBS was established at the national and states levels with some variation in supportive and core functions, performance of EBS as perceived by the assessment teams, state surveillance staff perception about EBS, and community volunteers’ performance and perception about EBS.

EBS and IBS are considered to be complementary and essential components of the national surveillance system [13]. This was not the case in Sudan; EBS at the national level is placed under the supportive activities’ unit of IBS, a situation that underestimates its usefulness and importance to an extent. Comparatively, EBS is equivalent to CEBS in some states. Factors contributing to the disproportionate weight given to IBS verses EBS are the weak links between the national EBS focal person and states and the long-time gap since the last training. A lack of understanding is considered one of the reasons for sub-optimal implementation of EBS capacities at states level as establishing an optimally functioning EBS system requires involvement of communities, health facilities, and partners [14]. Despite this fact, the situation in Sudan indicates that EBS is partially integrated in the existing system, and this is the case in other low- and middle-income countries where a review showed that all EBS systems were integrated into existing routine surveillance systems and pre-existing response structures to some extent [15]. However, potential EBS partners are not yet contributing to EBS in Sudan although they showed clear willingness to support the efforts of establishing the system. The One Health Approach- recently initiated at the national level in Sudan- could be used as a platform to enable capturing of events from human, animal, environmental, and other relevant sources [16, 17]. However, to move ahead with EBS system in any country depends on the country’s ability to review and adapt existing surveillance structures and to involve communities and partners [14]. Although electronic surveillance and internet-based systems proved their role in augmenting the traditional surveillance, particularly in detection, tracking and reporting of emerging infections [18], they have limited use for surveillance purpose in Sudan. Attempts to maximize the benefits from signals module are ongoing at national level and there is a plan to expand its function to states.

There was an uncertainty in the pathway between signal detection and action; this weakens the system, leading to delayed verification, risk assessment and response and hence lowers its performance. The other point to be mentioned here is that states report when there is a signal or event immediately, and the states also compiled all reported signals and events and send these to the Federal MOH as a monthly report. In the visited states, the assessment team found reports on each signal and event but few states showed a monthly EBS reports. This is potentially the cause of concern about the limited contribution of EBS to the general surveillance. Experience from the pilot project in Vietnam showed that presence of structured data and reports were valuable for the MOH to understand the function of the EBS program and the success and challenges of implementing EBS [19].

The EBS performance of the 6 visited states as scored by the assessment team revealed an overall higher score in many assessed items but states lagging behind and having concerns about presence of structured collaboration with partners and communities, presence of written organogram, roles and responsibilities, low capacity, limited access to internet and lack of supportive supervision. The lack of documented SOPs was recognized as a potentially compromiser to quality and consistency of practice in EBS and that implementation of SOPs, and continuous quality improvement processes are highly needed [20]. As well, case detection can be greatly improved through increased staff training and community engagement [21]. The EBS in Sudan could make use of the lessons learned from Vietnam EBS project which showed the value of supportive supervision, monitoring and evaluation to build sustainable system and to identify barriers to effective implementation [22].

The surveillance staff at states levels reported having a list of signals with standard definition, training community volunteers, and knowing the recommended time for verification. All respondents reported detecting signals/ events in the last year and conducting risk analysis. All the above-mentioned items were included in the EBS SOPs and guidelines, 2016, although comprehensive and updated EBS guideline is needed. Findings indicate the presence of a functioning CEBS system at states level with some limitations; a situation typical to what was reported about surveillance earlier in Khartoum State, the capital of Sudan [11].

The community volunteers are nominated by their communities, a factor that ensures the success of CEBS and increases the community’s acceptance, participation and trust [17]. Most of the community volunteers are satisfied with their tasks and are willing to continue. This is an ideal facilitating factor for the system and provides a better opportunity for sustainable CEBS. Other factors associated with better performance include regular training and supportive supervision from the national level. A study in Sierra Leone reported that training, guidelines and follow-up from supervisors, together with seeing results and having a role in helping their communities, were the main motivating factors [23]. The staff turnover and the gap between income and expenses were the biggest challenges that compromise the sustainability of the system. However, these compromisers can be mitigated by development of better mechanisms to publicize the role of volunteers, and improvement of recognition and appreciation schemes [24].

The assessment revealed that the strong points of EBS in Sudan included the presence of a functioning IBS, a trained and committed national team, strong guidelines and SOPs, dedicated volunteers, regular monthly reports, and functioning media scanning at the national level. The weakest points in the system were poor link between national and states level, disintegration of EBS components at national and state level, limited utilization of E-Systems at state level, under appreciation for the surveillance structure at national and state level, and a lack of strategic vision. Still there are opportunities for the development of resilient EBS system, and this is reflected in the partners’ willingness to participate and to support implementation. All these efforts could be compromised by high staff turnover, particularly at localities and states and by the COVID-19 pandemic. These findings highlighted the need for an EBS strategy, updated guidelines and training manuals.

## Limitations

The study was not able to assess the quality of EBS and its contribution in early detection and response as there was no registry for signals and events at state or national level or the information recorded was not complete. The small sample of the interviewed community volunteers showed their willingness and a satisfaction with their contribution to EBS, but it is not enough to conclude about the effectiveness of community volunteers. Further study needed to reflect the experience of CEBS.

## Conclusions and policy implication

There are efforts to improve EBS within Sudan as reflected by the national assignment of an EBS coordinator, development of guidelines and SOPs, and the training of EBS focal persons at states. These efforts are challenged by the underestimation of the EBS role by the national level and the misperception of it as an activity instead of a system. The pathway from “detection of signals/ events” to “data for action” is not clear and affects the timely use of data, delayed response, delayed integration and overall performance. The link between the national level and states is unstructured and driven by the need for reports. Despite the efforts to train states’ staff and frontline personnel, high staff turnover at state and localities attributed to political instability and low salary is still a big problem. Partners EBS in not functioning (the only exception is animal sector) in all states; however, there are partners willing to participate and support EBS. Electronic databases are ignored and not used or are even unknown especially at the state level. There is feedback coming from the national level to the states but not from the later to the localities and sentinel sites. Training, presence of trained staff and regular follow up are supporting factors, while staff turnover is the biggest challenge.

The study highlighted very important points that have implications of EBS policy. The FMOH needs to develop an EBS strategy, comprehensive guidelines and training manuals. Moreover, the ministry has to revise the list of signals/events, reporting format and recording procedures as well as to have in place a clear role and responsibilities of EBS actors.

## Data Availability

All data is available at the surveillance and Information Directorate (SID) at Sudan Federal Ministry of Health.

## Appendix

Table 2

**Table 2:** Performance of state (out of 10) in surveillance and EBS as scored by the visited teams, Sudan, 2021

**No**	**Statement**	N. Darfur	N. Kordofan	Northern	Khartoum	Sinnar	Gedarif	Average
**1**	State has a list of signals and events	10	10	10	10	10	10	10.0
**2**	State capacity to detect signals and events	5	4	4	10	8	6	6.2
**3**	State register signals and events regularly	5	5	5	8	8	8	6.5
**4**	State triages/verifies signals within 24 h	10	10	10	10	10	8	9.7
**5**	State reports signals and events to FMOH	4	3	3	10	10	10	6.7
**6**	There is structured collaboration with partners	1	1	1	8	1	1	2.2
**7**	There is an EBS at state level	8	1	1	8	0	0	3.0
**8**	There is CEBS at state level	8	6	6	4	7	7	6.3
**9**	EBS is part of HEEC structure	7	0	0	8	6	7	4.7
**10**	State assesses risk within 48 h	10	10	10	10	10	8	9.7
**11**	State responds within 48 h	7	10	10	10	10	8	9.2
**12**	HEEC at state level has a functional structure	7	2	0	8	10	10	6.2
**13**	HEEC has written roles and responsibilities	7	0	0	0	0	0	1.2
**14**	State has all national guidelines and SOPs	5	3	7	8	7	10	6.7
**15**	Staff at state and localities are trained	1	7	5	7	3	3	4.3
**16**	State has a budgeted plan	10	10	10	0	10	10	8.3
**17**	There is a known budget for surveillance	7	10	2	0	10	10	6.5
**18**	State has free access to internet	3	0	10	6	0	0	3.2
**19**	State is part of the national HEEC network	8	10	10	9	8	8	8.8
**20**	State has a plan and checklist for supervision	2	10	10	10	10	5	7.5
**21**	State conducts supervision regularly	1	1	1	8	2	2	2.5

Table 3

**Table 3:** Detection and reporting of signals and events and involvement of non-health sectors and partners (*n* = 53)

Variables	Description	Frequency	%
List of syndromes and events and system for capturing	Having EBS list of syndromes and events	31	58.5
	Having definition of syndromes and events	32	60.4
	Having community-based system	42	79.2
	Having a system or mechanism to capture unusual, unexpected or new event from non-routine sources	40	75.5
Information on unusual, unexpected or new signals/ events collected using	Call centre	48	90.6
	Social media	37	69.8
	Newspapers	21	39.6
	Emails	22	41.5
	Radio/ TV	27	50.9
	Volunteers	48	90.6
	Health care workers	46	86.8
Involvement of different actors in reporting of unusual, unexpected or new signals/ events	Laymen	39	73.6
	Volunteers	46	86.8
	Health care workers	46	86.8
	Veterinary personnel	26	49.1
	Officials governmental sector	37	69.8
Level involved in detection of unusual, unexpected or new signals/ events	Community level	51	96.2
	Health facility level	53	100.0
	Locality level	46	86.8
	State level	45	84.9
	National level	37	69.8
Degree of contribution of veterinary sector	Not contributed	13	24.5
	Contributed on regular basis	9	17.0
	Contributed but not on regular basis	31	58.5

## Abbreviations

CEBS: Community event-based surveillance
COVID-19: Corona Virus Disease 2019
EBS: Event-based surveillance
ECC: Emergency call centres
EOC: Emergency Operation Centre
EWAR: Early Warning Alert and Response
FGD: Focus Group Discussions
FMOH: Federal Ministry of Health
HEEC: Health Emergency and Epidemic Control
HMS: Hotline and Media Scanning
IBS: Indicator-based surveillance
IHR: International Health Regulations
ITS: Incidence Tracking System
MoH: Ministry of Health
NGOs: Non-Governmental Organization
OSM: Online Signal Module
PEBS: Partner-Based Surveillance
POE: Point of Entry
SID: Surveillance and Information Department
SOP: Standard Operation Procedures
SPSS: Statistical Package for Social Science
UN: United Nation
WHO/EMRO: World Health Organization/ East Mediterranean Office
